# Uterine transplantation in transgender women

**DOI:** 10.1111/1471-0528.15438

**Published:** 2018-09-20

**Authors:** BP Jones, NJ Williams, S Saso, M‐Y Thum, I Quiroga, J Yazbek, S Wilkinson, S Ghaem‐Maghami, P Thomas, JR Smith

**Affiliations:** ^1^ West London Gynaecological Cancer Centre Hammersmith Hospital Imperial College NHS Trust London UK; ^2^ Department of Surgery and Cancer Imperial College London London UK; ^3^ Department of Politics, Philosophy and Religion Lancaster University Lancaster UK; ^4^ Lister Fertility Clinic The Lister Hospital London UK; ^5^ The Oxford Transplant Centre The Churchill Hospital Oxford University Hospitals NHS Trust Oxford UK; ^6^ Brighton Gender Clinic Nuffield Health Hospital Brighton UK

## Introduction

Gender dysphoria, defined as the persistent discomfort with one's gender identity or biological sex, affects between 0.5 and 1.4% of adult males.^1^ Treatment aims at congruence, to allow those who experience it to find comfort within their gendered self, which optimises psychological wellbeing and self‐fulfilment.^2^ Although many experiencing gender dysphoria require partial treatment or social transition, others only find comfort following surgical intervention to change their external genitalia and sexual characteristics. Traditionally, infertility has been an unfortunate consequence of the realignment of a transgender person's body with their gender identity.

Following a successful clinical trial investigating uterine transplantation (UTx) in Sweden, resulting in eight live births so far,^3^ UTx appears to be a viable therapeutic option for women with absolute uterine factor infertility (AUFI). More than 42 UTx procedures have now been performed globally, and at least 12 live births have been reported. Following the establishment of the International Society of Uterine Transplantation (ISUTx), and the formation of research teams globally, it is anticipated that UTx will make the transition from research to clinical care in the future. Following these developments, speculation has escalated regarding the possibility of performing UTx in male to female (M2F) transgender women, which would enable them to gestate and give birth to their own children.^4^

Ethically, the consideration of performing UTx in transgender women is primarily motivated by the considerations of justice and equality. Like all women, psychological harm may arise secondary to a mismatch between reproductive capacity and aspiration. Transgender women have AUFI, and therefore they cannot experience gestation, which may play an integral role in the expression and consolidation of a female identity,^5^ and is considered by many to constitute a transformative experience.^6^ Legally, under the Equality Act (2010) transgender people are afforded explicit protection from both direct and indirect forms of discrimination through the characterisation of ‘gender reassignment’ as a protected characteristic. As such, M2F transgender women cannot be subjected to discrimination on the basis of this characteristic. Subsequently, if UTx becomes an established treatment option for women with AUFI, UK and EU legislation would make it legally impermissible to refuse to perform UTx in transgender women solely because of their gender identity. Performing UTx in this population, however, raises a number of anatomical, physiological, fertility, and obstetric considerations. The aim of this manuscript is to discuss these factors and provide an initial framework for assessing the feasibility of UTx in M2F transgender women.

## Anatomical considerations

Uterine transplantation entails the transplantation of the uterus, including the cervix, a cuff of vagina, the surrounding ligamentous and connective tissues, as well as the major blood vessels to the level of the internal iliac vessels. The uterus is then placed orthotopically in the pelvis of the recipient, where it is structurally supported using the uterosacral, round and broad ligaments laterally, the bladder peritoneum anteriorly, and the vagina and paravaginal tissues inferiorly.

### Vascular anastomosis

Most UTx procedures performed to date have achieved revascularisation through bilateral internal iliac artery to external iliac end‐to‐side anastomoses. To determine the plausibility of retrieving a graft from a woman, with subsequent implantation into a natal male pelvis, the intersex differences in pelvic vascular anatomy require consideration. Fătu et al.^7^ assessed the morphometry of the internal iliac arteries between sexes, and concluded there was no difference in internal iliac artery length, with a mean length of 49 mm. However, the calibre of the vessels was noted to be 1.6 mm wider in females than in males (6.2 versus 7.8 mm).^7^ Although a significant funnelling effect could predispose to thrombosis, this difference could be negated by anastomosing further proximally, where the vessels are similarly sized. With regard to the external iliac arteries, data from lower limb angioplasties show there is no significant difference in external iliac artery calibre between sexes.^8^

### Vaginal anastomosis

In all UTx cases performed to date, the recipient's vagina has been anastomosed to a vaginal cuff, of varying length, which is retrieved as part of the graft. This restores reproductive anatomy and allows physiological excretion of discharge and menstruation, but also allows direct visualisation of the cervix and access to take biopsies, which is the only reliable way to detect rejection following UTx.^9^ In the M2F transgender model, it would therefore only be possible following gender reassignment surgery (GRS), which traditionally includes orchidectomy, penectomy, clitoroplasty, and labiaplasty, with the subsequent creation of a neovagina. The inverted penile skin flap is the standard technique for neovagina creation,^10^ to line a newly created space between the bladder/prostate and the rectum. However intestine,^11^ or pelvic peritoneum,^12^ have also be utilised, particularly in cases with penoscrotal hypoplasia, which can be an iatrogenic consequence of feminising hormones. However, the absence of a physiologically functioning vaginal mucosa may be problematic. The vagina is lined by multiple layers of stratified squamous epithelium, the top layer of which removes adherent micro‐organisms by desquamation into the vaginal lumen.^13^ Vaginal epithelium also facilitates the recognition of pathogens and stimulates production of antimicrobial peptides and pro‐inflammatory cytokines.^14^ These protective mechanisms contribute to the creation of a commensal microflora, predominantly consisting of lactobacilli, which provides an optimal physiological environment to prevent infection and maintain pregnancy. In M2F transgender women, the pH in penile skin‐lined neovaginas is elevated, owing to an inability to support the growth of acidic lactobacilli, with colonisation of bacteria from skin or intestinal microfloras instead.^15^ Following M2F transgender UTx, the presence of a skin or intestinal neovagina, in the context of immunosuppression, may increase susceptibility to recurrent neovaginal infections and create a hostile environment that may be incapable of sustaining pregnancy. This was exemplified in the UTx case performed in Turkey in a recipient with an intestinal neovagina.^16^ Despite multiple embryo transfers and at least six early pregnancy miscarriages, she has yet to achieve a live birth.^17^ Moreover, the only woman in the Swedish series to have not yet given birth following successful UTx, despite suffering at least five miscarriages, has a skin neovagina.^18^ Although it appears the absence of a physiologically functioning vagina is detrimental, albeit to a currently unquantified extent, small numbers of live births have been reported in women with skin neovaginas,^19^ including two following UTx in the Swedish series,highlighting that successful pregnancy is possible.

**Figure 1:**
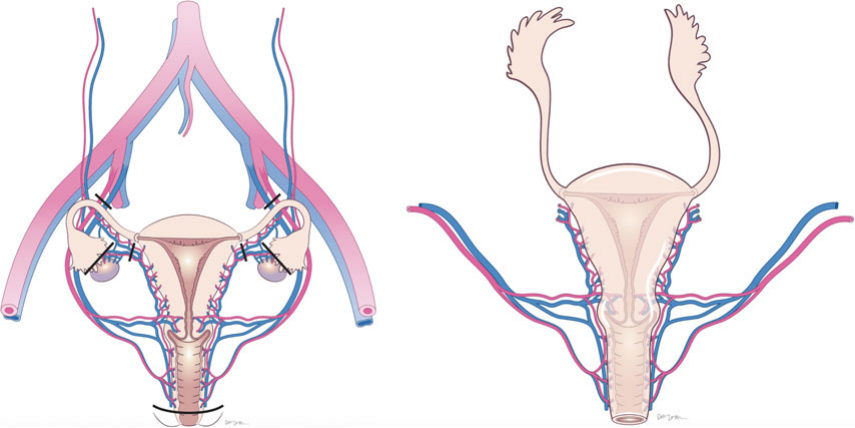
Proposed utero‐vaginal retrieval operation with incision markings (left) and the utero‐vaginal transplant graft following retrieval, including vascular pedicles (right).

To overcome this anatomical hindrance in M2F transgender women, a utero‐vaginal transplant could be performed, utilising as much donor vagina as possible, en‐bloc with the uterus (Figure 1). This would be achievable using a similar technique to that employed at radical hysterectomy, with preservation of the vaginal branches of the uterine vessels. This would necessitate retrieval from deceased donors, excluding female living donors. An alternative donor pool may be female to male (F2M) transgender men undergoing hysterectomy and vaginectomy, although the increased radicality of the hysterectomy may not be acceptable. However, recent evolution of the surgical technique, following cases in China^20^ and Dallas,^21^ has potentially significantly reduced donor risk. The modified technique utilises venous drainage of the graft via the ovarian or utero‐ovarian veins, as opposed to the unpredictable and tortuous uterine venous plexus which leads to the internal iliac veins. This negates the need for the complex and time‐consuming ureteric dissection away from the uterine veins, reducing surgical risk. Moreover, it reduces operative times from 12 hours to 4–5 hours, which in turn decreases potential venous thromboembolism (VTE) risk. This dissection also favours minimally invasive retrieval techniques, which should enhance recovery and reduce potential morbidity further. As most F2M transgender men will also undergo bilateral oophorectomy, whereas the ovaries it would not be transplanted, it would allow the retrieval of elongated ovarian vascular pedicles to facilitate the implantation.

**Figure 2:**
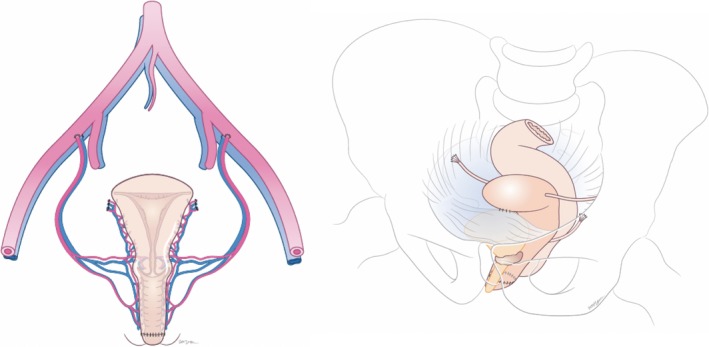
Male to female transgender anatomy following utero‐vaginal transplantation demonstrating vascular and vaginal anastomoses (left), and ligamentous support following implantation (right).

The graft for implantation, using deceased donors, is displayed in Figure 1. This would be anastomosed to the recipient's neovagina as shown in Figure 2. Although prostatectomy is not routinely undertaken in GRS, the oestrogenised environment in transgender women causes prostatic atrophy,^22^ which should not cause a structural hindrance in UTx.

### Ligamentous support

The ligamentous and soft tissue support is provided anteriorly by approximating donor and recipient bladder peritoneum. This technique is directly transferable to the M2F transgender model. Postero‐laterally, the broad and uterosacral ligaments from the donor are connected to the pelvic side wall and uterosacral remnants, respectively, in the recipient. Although M2F transgender women do not have uterosacral remnants, this could be overcome by a more radical ligamentous retrieval, with subsequent anastomosis to the recipient paraneovaginal region (Figure 2).

## Hormonal factors

Exogenous estrogen optimises the development of female secondary sexual characteristics, whereas anti‐androgens, such as spironolactone or finasteride, minimise male features. Progestogen is not routinely administered, as its role in feminisation remains unclear.^23^ However, considering that unopposed estrogen is a significant risk factor for endometrial hyperplasia and endometrial carcinoma in post‐menopausal women,^24^, ^25^ progestogen supplementation would become essential in transgender women following UTx.

Continuous hormone replacement therapy (HRT) is the usual regimen prescribed in M2F transgender women, but sequential HRT would be more appropriate following UTx in transgender women. Not only is withdrawal bleeding an important sign of graft function, but it is intrinsically part of being female and therefore contributes to gender identity, which may have psychological benefits.^26^

Graft thrombosis is one of the most common serious complications following organ transplantation. Although oral estrogen was previously implicated in an increased VTE risk in M2F transgender women,^27^ this was later attributed to the use of ethinylestradiol, a particularly thrombogenic estrogen that is no longer in routine use. A subsequent study on 2236 M2F transgender individuals reaffirmed this, with no additional risk of VTE seen in those receiving different hormone therapy.^28^

## Fertility considerations

Fertility preservation should be discussed in all M2F cases prior to the commencement of hormone therapy or contemplation of GRS. M2F transgender women can preserve their fertility prior to transition using sperm cryopreservation, with subsequent *in vitro* fertilisation (IVF) or intrauterine insemination (IUI) in a female partner or surrogate.

Following UTx, embryo transfer should not be attempted until at least 6 months postoperatively, to allow healing and stabilisation of immunosuppression. Achieving pregnancy may be feasible utilising hormone regimens that have been used with success in women with premature ovarian insufficiency or following physiological menopause.^29^ Following the withdrawal bleed on sequential combined HRT, estrogen supplementation should be commenced to stimulate the endometrium. Once >7 mm in thickness, progesterone should be supplemented to maintain the endometrial lining for implantation. A single embryo can then be transferred into the uterus. Multiple embryo transfers should be avoided owing to the additional risks associated with multiple gestations. All women should have previously undergone orchidectomy, with resultant low testosterone levels. However, if anti‐androgens such as finasteride or spironolactone are being taken, these should also be stopped in advance of fertility treatment, owing to their teratogenic potential.^30^, ^31^

## Obstetric considerations

Male pelvises differ from their female counterparts, to an extent that they can be used to determine gender at autopsy.^32^ This dimorphism has evolved as a consequence to sex‐specific selection pressures.^33^ Natal males need a pelvis suitable for bipedal locomotion, whereas the female pelvis must also accommodate a fetus during pregnancy and be adequately capacious for childbirth.^33^ Although most skeletal measurements are larger in males than females, the true pelvis of the female has evolved to become larger and broader.^34^ This dimorphism is most marked at the antero‐posterior diameter of the pelvic inlet, the transverse diameter of the midplane between the ischial spines, and the transverse diameter of the pelvic outlet.^34^ Moreover, whereas the pelvic inlet is oval‐shaped in females, it is heart‐shaped in males. These intersex differences in pelvic morphology would predispose M2F females after UTx to cephalopelvic disproportion should labour be attempted. However, as the requisite mode of delivery in women following UTx is caesarean section, owing to concerns regarding the mechanical strain of labour, this should also be the case in M2F transgender women.

Sexual dimorphism arises predominantly due to the outcome of gender‐determined autosomal genes, which are regulated by sex‐specific hormones and influenced by hormone receptor sensitivity.^35^ This dimorphism has been demonstrated in the pelvis, where despite similar growth patterns throughout childhood, it is not until puberty when the growth trajectory increases in females, and not until the late twenties when the pelvis attains the most favourable obstetric dimensions. As such, if M2F transgender women undergo hormone therapy at a young enough age, they may develop similar pelvic morphology to natal females. Although there is no evidence in the context of M2F transgender women, the opposite effect has been demonstrated in female to male (F2M) transgender men, where a biometric analysis of pelvic characteristics after the onset of hormone therapy revealed evidence of ‘masculinisation’.^36^

## Conclusion

Despite a number of anatomical, hormonal, fertility, and obstetric considerations that require consideration, there is no overwhelming clinical argument against performing UTx as part of GRS. However, the increased radicality associated with the retrieval operation, including a longer vaginal cuff and more extensive ligamentous dissection, potentially necessitates the use of deceased donors. Alternatively, F2M transgender men may offer an alternative donor pool should they accept the increased risk compared with standard hysterectomy. Prior to undertaking UTx in transgender women, further research should be undertaken including cadaveric retrieval and implantations to assess the feasibility of the anatomical considerations discussed herein. Furthermore, it is recommended that animal studies are revisited to identify potential unknown risks and determine whether genetic males can successfully conceive and maintain pregnancy.

The reproductive aspirations of M2F transgender women deserve equal consideration to those assigned female at birth and, subject to feasibility being shown in the suggested areas of research, it may be legally and ethically impermissible not to consider performing UTx in this population.

### Disclosure of interests

None declared. Completed disclosure of interest forms are available to view online as supporting information.

### Contribution to authorship

The article was conceived and written by BPJ. NJW helped write the article and reviewed the final draft. SS, MYT, IQ, JY, SW, SGM, PT, and JRS reviewed and contributed to the manuscript.

### Details of ethics approval

Not applicable.

### Funding

This work was supported by the Wellcome Trust [097897/Z/11/Z].
